# The geographic distribution and community correlates of electronic cigarette use in Canada

**DOI:** 10.17269/s41997-025-01084-8

**Published:** 2025-07-25

**Authors:** Adam M. Lippert, Daniel J. Corsi

**Affiliations:** 1 Department of Sociology, University of Colorado, Denver, CO USA; 2 Department of Obstetrics, University of Ottawa, Ottawa, ON Canada; 3 Children’s Hospital of Eastern Ontario Research Institute, Ottawa, ON Canada; 4 Faculty of Medicine, University of Ottawa, Ottawa, ON Canada

**Keywords:** Nicotine, Electronic cigarettes, Smoking, GIS, Nicotine, Cigarettes électroniques, Fumer, Système d’information géographique

## Abstract

**Objectives:**

Electronic cigarettes and other novel electronic nicotine delivery systems (ENDS) have grown rapidly in popularity and accessibility. In this study, we compiled a large sub-provincial dataset on smoking and vaping behaviour in Canada to inform targeted surveillance and prevention.

**Methods:**

Twelve national-level survey datasets were concatenated. Multilevel models were used to derive precision-weighted estimates of census division-level smoking and ENDS use prevalence, adjusted for age, sex/gender, and data source. We developed visualizations of the geography of smoking and ENDS use across Canada and used Census Divisions for spatially explicit correlational analyses of community characteristics associated with vaping.

**Results:**

The age- and sex-adjusted prevalence of past-month (i.e., current) ENDS use in Canada was 4%, with higher estimates observed in several Atlantic provinces: New Brunswick (5.6%), Prince Edward Island (4.8%), Nova Scotia (4.7%), and Newfoundland and Labrador (4.5%) followed by Manitoba (4.1%). Estimates for the remaining provinces were below 4%. The prevalence of ENDS use varied considerably across CDs, even in provinces where vaping was generally uncommon. Suburban and exurban communities in Ontario and Quebec demonstrated especially high ENDS use. Spatial analyses revealed select correlations with community factors such as economic composition.

**Conclusion:**

Sub-provincial data revealed geographical variability in ENDS use across Canada. Localized surveillance and prevention efforts may be improved by considering the community features associated with high rates of use, and benchmarking regional regulations on the advertising and sales of ENDS products to communities with lower estimated rates of use.

## Abstract (fr)

**Résumé:**

**Objectifs** Les cigarettes électroniques et autres nouveaux dispositifs électroniques d’administration de nicotine (DÉAN) sont de plus en plus populaires et accessibles. Dans cette étude, nous avons compilé un vaste ensemble de données sous-provinciales sur le tabagisme et le vapotage au Canada pour éclairer la surveillance et la prévention ciblées.

**Méthodes:**

Nous avons concaténé 12 ensembles de données d’enquête nationales. À l’aide de modèles multiniveaux, nous avons dérivé des estimations, pondérées selon leur précision, de la prévalence du tabagisme et de l’usage des DÉAN par division de recensement, que nous avons ajustées selon l’âge, le sexe/le genre et la source de données. Nous avons mis au point des visualisations géographiques du tabagisme et de l’usage des DÉAN au Canada et utilisé les divisions de recensement pour mener des analyses corrélationnelles spatialement explicites des caractéristiques communautaires associées au vapotage.

**Résultats:**

La prévalence rajustée selon l’âge et le sexe de l’usage des DÉAN au Canada au cours du mois précédent (autrement dit, la prévalence actuelle) était de 4%; des estimations plus élevées ont été obtenues dans plusieurs provinces de l’Atlantique – au Nouveau-Brunswick (5,6%), à l’Île-du-Prince-Édouard (4,8%), en Nouvelle-Écosse (4,7%) et à Terre-Neuve-et-Labrador (4,5%) – ainsi qu’au Manitoba (4,1%). Les estimations pour les autres provinces étaient inférieures à 4%. La prévalence de l’usage des DÉAN variait considérablement selon la division de recensement, même dans les provinces où le vapotage était peu commun en général. Les communautés suburbaines et exurbaines de l’Ontario et du Québec affichaient un usage des DÉAN particulièrement élevé. Les analyses spatiales ont mis au jour des corrélations avec des facteurs communautaires comme la composition économique.

**Conclusion:**

Les données sous-provinciales ont révélé la variabilité géographique de l’usage des DÉAN au Canada. Il serait possible d’améliorer les démarches de surveillance et de prévention locales en tenant compte des caractéristiques communautaires associées aux taux d’usage élevés et en comparant la réglementation régionale de la publicité et des ventes de DÉAN dans les communautés où les taux d’usage estimatifs sont moins élevés.

## Introduction

Since their introduction to North American markets in 2007, electronic cigarettes (e-cigarettes) and other novel nicotine-based products have witnessed a surge in popularity and accessibility. These products, collectively known as electronic nicotine delivery systems (ENDS), heat liquids, often containing nicotine, flavourings, and carrier components, to produce an inhaled aerosol. Initially developed as a less harmful alternative to traditional smoking and a smoking cessation aid, their effectiveness in this regard remains to be determined (Lippert, 2015). At the same time, the usage of these products has quickly increased—particularly among tobacco-abstinent youth and young adults (Erhabor et al., 2023). Canadian data reveal a significant increase in past-30-day ENDS use among youth under 20 between 2013 and 2022, from 4 to 14%. Notably, over two-thirds (69%) of young ENDS users aged 15 to 19 years and over half of users aged 20 to 24 years (62%) have never smoked (Statistics Canada, 2022a). In the United States (USA), data from multiple large-scale surveys suggest that current ENDS use among high schoolers approached 30% in 2019 (Boakye et al., 2023). Data from the 2021–2022 Canadian Student Tobacco, Alcohol, and Drugs Survey show that 29% of students in grades 7–12 had ever used an e-cigarette, with 17% reporting ENDS use in the past 30 days (Statistics Canada, 2022b).

The rapid uptake of vaping, especially among non-smoking youth and young adults, raises serious public health concerns. Potential long-term consequences of vaping include a higher likelihood of initiating cigarette smoking (Yoong et al., 2021), cannabis use (Sun et al., 2022), and numerous respiratory injuries resulting from contact with e-cigarette aerosols that are rich in carcinogenic compounds jeopardizing pulmonary health. These compounds include heavy metals, aldehydes, and nitrosamines (Traboulsi et al., 2020)—compounds associated with cellular damage, oxidative stress, and respiratory impairments (Lerner et al., 2016; Yogeswaran et al., 2021). Other adverse outcomes associated with ENDS use include damage to teeth and gums (Huilgol et al., 2019), impaired neurological development (Ruszkiewicz et al., 2020), and heightened vulnerability to infectious diseases (Pushalkar et al., 2020).

In light of the health risks posed by ENDS use, surveillance and prevention efforts must be informed by the known correlates of vaping—including those found in the community environment. While health behaviours vary from place to place and are patterned by socio-environmental features (Johansen et al., 2006), geographically based data on ENDS use in Canada are notably scarce. Various systematic reviews have explored the socio-ecological factors influencing ENDS use (Kong et al., 2024), though few include studies across Canadian communities. Notably, sub-provincial data on ENDS usage have not been publicly disclosed or utilized, although the necessary geographic identifiers for such analyses exist within national health surveys. Preceding the 2018 *Tobacco and Vaping Product Act*, Canadian provinces and territories implemented diverse regulations to govern ENDS use and accessibility. Years later, these differing regulations persist, impacting youth usage rates and potentially contributing to regional disparities in ENDS consumption nationwide (Statistics Canada, 2017a). While ENDS use among youth has increased in all provinces and territories, this increase has been more moderate in provinces with stricter legislation—a pattern that may be attributable to differences in perceived harm and accessibility of vaping among youth across provinces (Montreuil et al., 2017).

The current study responds to the lack of sub-provincial data on vaping in Canada via three main aims: (1) apply multilevel regression modelling and precision-weighted estimation (PWE) to derive *empirical* estimates of smoking and ENDS use across sub-provincial geographies (i.e., Census Divisions [CDs]) in Canada; (2) map rates of smoking and ENDS use across CDs; (3) use spatially explicit correlational analysis to identify community characteristics associated with high and low rates of ENDS use. By assembling this data resource and assessing sub-provincial variation in ENDS use, the current study informs prevention and harm reduction efforts while helping to harmonize policies and regulations that vary across Canadian provinces.

## Methods

### Data

Source data span ten original datasets concatenated and used via the methods described in the forthcoming section. A description of the source data is provided below (see Appendix for additional details):

#### Canadian Tobacco Alcohol and Drug Survey (CTADS)

Operated as a national telephone survey that excluded the territories, the CTADS tracked the usage of tobacco, e-cigarettes, alcohol, and drugs across Canada. Conducted every 2 years, data are available spanning from 2013 to 2017. The survey is aimed at Canadians who are 15 or older and reside in the provinces, achieving an average response rate of approximately 77.1%. Between 2013 and 2017, the number of respondents ranged from 14,565 to 16,349. Among the various topics addressed in the CTADS are facets of ENDS usage, such as where users acquire e-cigarettes, the employment of ENDS for quitting smoking, perceptions of risk, and motivations for beginning ENDS usage. In 2019, CTADS was revised to be conducted as two new independent surveys: the Canadian Tobacco and Nicotine Survey (CTNS) and the Canadian Alcohol and Drugs Survey (CADS).

#### Canadian Tobacco and Nicotine Survey (CTNS)

The CTNS surveys consumption of tobacco and nicotine products, targeting those 15 years and older residing in any province. The survey does not include individuals who are part of collectives, living in reserves, or are institutionalized. In 2019, the survey was completed by 8614 respondents; this number was 8112 in 2020 and reached 9908 in 2021. The CTNS measures smoking, utilization of various tobacco items, vaping, and consumption of alcohol and cannabis.

#### Canadian Alcohol and Drugs Survey (CADS)

Targeted at Canadians aged 15 and above, the CADS excludes individuals who live in any of the three Canadian territories, are institutionalized, or reside on First Nations reserves. In 2019, the survey garnered responses from more than 10,000 individuals, covering topics such as alcohol intake, driving while intoxicated, and cannabis and illicit drug use. Additionally, the CADS gathers data on alcohol consumption during pregnancy from respondents who have experienced pregnancy within the preceding 5 years.

#### Canadian Community Health Survey (CCHS)

The CCHS is an extensive yearly health assessment covering numerous topics. This includes in-depth inquiries about smoking habits and e-cigarettes (beginning in 2015). The CCHS is a large-scale survey designed to produce reliable health indicators at the sub-provincial level, such as Census Divisions. The survey focuses on Canadians who are 12 years of age or older, drawing in roughly 65,000 participants each year. However, it does not encompass individuals living on Indigenous Reserves or Crown Land, those residing in institutions, full-time members of the Canadian Armed Forces, and inhabitants of some isolated areas. The survey’s methodology ensures robust estimates at sub-provincial levels through a multi-stage sampling approach, yielding [nearly] equal weighting to each region and province (Statistics Canada, n.d.). The CCHS captures participants’ use of ENDS in the past month.

Using restricted-access versions of each dataset, we can map estimates to standard Census Geographics at provincial and sub-provincial levels through postal code identifiers. The Postal Code Conversion File (PCCF), conceived by Statistics Canada (Statistics Canada, 2017a, 2017b. Catalogue No. 92–154-X), along with an affiliated SAS program, translates respondents’ six-digit postal codes to Census Dissemination Areas (DA). With this program, respondents are associated with their DA residence, allowing us to identify the related Census Division. Our analysis primarily utilizes the Census Division level for privacy and adherence to data aggregation and release guidelines. These divisions are Canada’s second-tier geographies, situated just beneath the provinces (Statistics Canada, 2017b). While their primary function is for statistical enumeration, they represent regional divisions, historic counties, or conglomerates of urban or rural municipalities in various provinces. Previous studies show that health outcomes fluctuate across Census Divisions, emphasizing their role in tracking disparities in health outcomes and associated risks. Databases used in this study were accessed via the Canadian Research Data Centre Network (CRDCN) and the Ottawa-Outaouais Research Data Centre (ORDC).

The concatenated dataset was limited to only those respondents with valid geo-identifiers in the restricted-use version of the source data (*n* = 722 missing geo-identifiers). With this inclusion criterion, we achieved an analytic sample of 225,991 individuals from all 10 provinces with a median of 304 individuals nested within each of the 293 CDs represented in the study. Across all source data, individual items tapping nicotine use outcomes were generally consistent, with each survey having at least two nearly identical items assessing lifetime and past-month smoking and ENDS use. The CCHS used a multi-stage stratified sampling design, while the CTNS and CADS used stratified random sampling. Thus, we adjust all empirical estimates for data source and survey year.

### Variables

For area-level primary outcomes, we assessed the percentage of individuals within a CD who reported using ENDS in the past month. An analogous measure was calculated for current smoking. Given that past-month usage often signifies active or ongoing consumption, our analysis centred on past-month nicotine and e-cigarette usage. While calculating use rates across these areas, we incorporated basic controls for the data source (including survey type and year) and the age and sex/gender of each survey respondent. Our analysis incorporated survey weights from the individual datasets, adjusting them for both pooled surveys and multilevel models (Carle, 2009; Corsi, 2012).

Utilizing the 2016 Canadian Census, which represents the midpoint of the 2013–2019 period within which our source data were collected, we assessed the covariation of Census division-level factors with e-cigarette usage rates. These community correlates, sourced from the 2016 Census master file and structured at the CD level, encompassed median age, percentage of visible minorities (with non-Caucasian categories for Chinese, Black, South Asian, and others), Indigenous identity composition (including First Nations, Metis, or Inuit), educational attainment (i.e., those holding a four-year college degree), median income, percentage in low income (after-tax measures), unemployment rates for those 15 and older, a female labour opportunity index (reflecting the female-to-male ratio in ‘white collar’ jobs as per the NOC, 2016), and the index of concentration at the extremes (ICE), which gauges the ratio of households in the top and bottom income quintiles (Krieger et al., 2016).

### Approach

Using data on CD-level nicotine use, data source (survey product and year), and minimal adjustments for respondent age and sex/gender, we derived precision-weighted estimates (PWE) from random-intercepts multilevel logistic regression models (Eq. 1) for a complete dataset of CD-level smoking and ENDS use. This process utilized Stata’s melogit command and unfolded over several steps. First, we retained CD-level predicted rates of combustible and electronic cigarette use with the adjustments previously described. We then applied PWE to these estimates via a “shrinkage” estimator that attenuates the least precise estimates towards the grand mean of the full sample following Eq. 2. In this equation, the shrinkage estimator is given by multiplying a community-specific residual ${{\boldsymbol{r}}}_{{\boldsymbol{j}}}$ by the ratio of a community-level variance component (${{\boldsymbol{\sigma}}}_{{\boldsymbol{u}}0}^{2}$) and the total variance of the estimate (${{\boldsymbol{\sigma}}}_{{\boldsymbol{u}}0}^{2}+{{\boldsymbol{\sigma}}}_{{\boldsymbol{e}}0}^{2}$) adjusted for each community-specific sample size (${{\boldsymbol{n}}}_{{\boldsymbol{j}}}$). Intuitively, this attenuates—or *shrinks*—the empirical estimates of all outcomes towards the grand mean in the sample, particularly for communities that provided relatively few survey respondents. Estimation procedures yielded no convergence issues and model parameters indicated good fit with the observed data. Point estimates and confidence intervals for both nicotine outcomes across all CDs may be found online: https://github.com/lippertam/CJPH_LIPPERT_CORSI.

1 $${y}_{ij}={\beta }_{0}+{\beta }_{1}{x}_{1ij}+({u}_{0j}+{e}_{0ij})$$

2 $${\widehat{u}}_{0j}={r}_{j}\times \frac{{\sigma }_{u0}^{2}}{{\sigma }_{u0}^{2}+{\sigma }_{e0}^{2}/{n}_{j}}$$

### Analysis

Using the empirically estimated dataset, spatially explicit methods are then applied to assess variation in the prevalence of smoking and ENDS use in relation to differences in compositional attributes across CDs. We begin by visualizing the geography of smoking and ENDS use across Canada, using CDs as approximations for communities. We then provide results from spatially explicit correlational analysis of community characteristics and their association with e-cigarette use rates. This is achieved with Lee’s *L* correlation coefficient, given by Eq. 3:

3 $${L}_{X,Y}=\frac{{\Sigma }_{i}[{(\Sigma }_{j}{w}_{ij}({x}_{j}-\overline{x }))\bullet ({\Sigma }_{j}{w}_{ij}({y}_{j}-\overline{y }))]}{\sqrt{{\Sigma }_{i}({x}_{i}-\overline{x }{)}^{2}}\sqrt{{\Sigma }_{i}({y}_{i}-\overline{y }{)}^{2}}}$$

…where *w*_*ij*_ is a row-standardized spatial weight for every *i*^th^ Census Division in each *j*^th^ network of nearby Census Divisions. This equation is essentially an amalgam of Pearson’s *r* and Moran’s *i* statistics to produce *L*_*X,Y*_—a measure of both bivariate correlation and spatial autocorrelation ranging from − 1 (indicating an inverse relationship between two variables) to + 1 (indicating a positive relationship).

A fundamental parameter in Eq. 3 is the spatial weight derived from the spatial neighbourhoods in which nearby Census Divisions are grouped. Following prior guidance (Chi & Zhu, 2020), these “spatial contiguity matrices” were identified via a series of trials to determine which specification yielded the highest spatial clustering of e-cigarette use rates—which in this case was a three-nearest-neighbour contiguity matrix, yielding a Moran’s *i* value of 0.23 calculated via 1000 Monte Carlo simulations. While significant, by most conventional standards, this value indicates only modest spatial clustering of vaping rates.

### Privacy

Throughout all analyses, small cell sizes that may pose a risk to identification and privacy were suppressed or merged with neighbouring units to meet the confidentiality requirements of the Statistical Act.

## Results

**Fig. 1:**
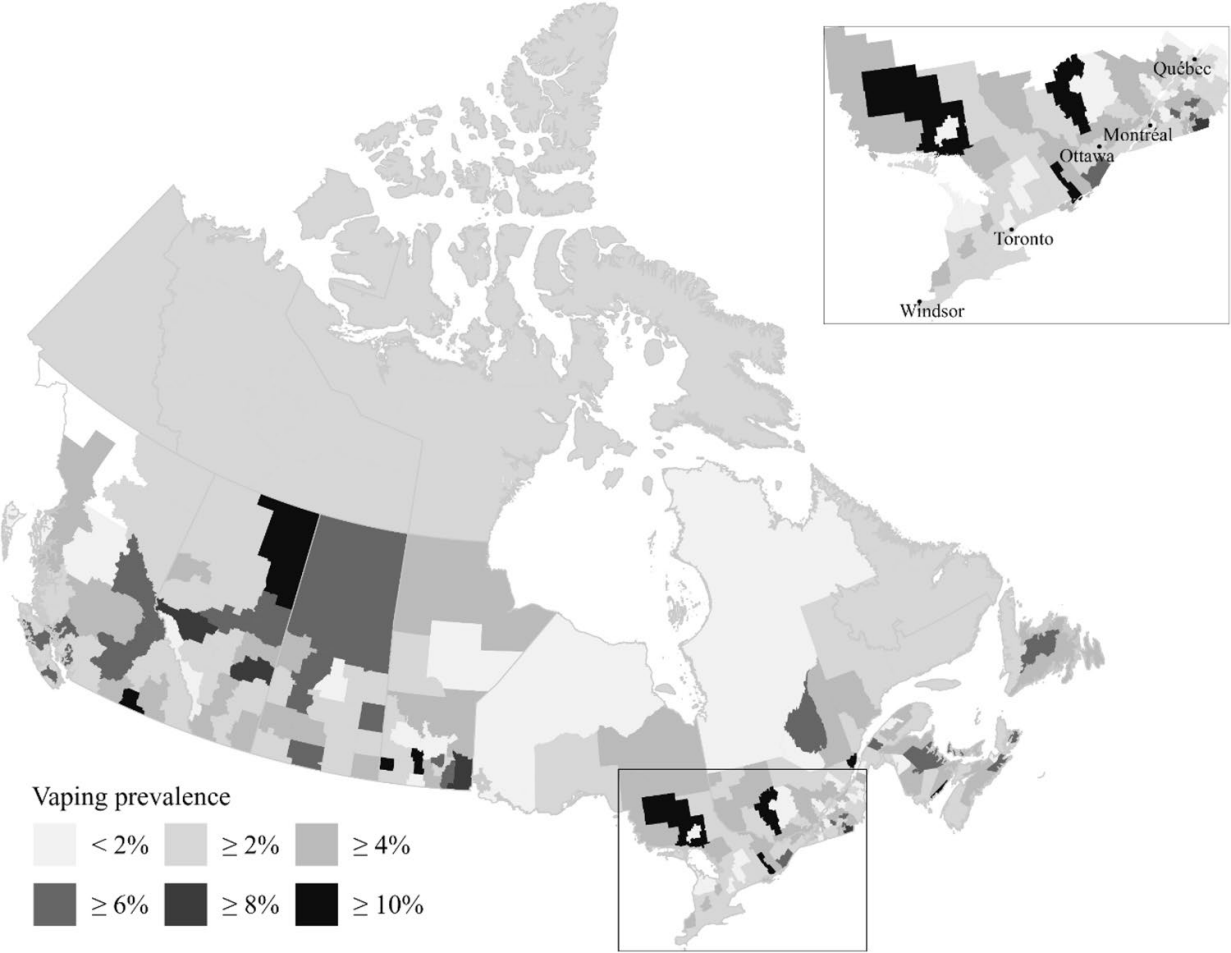
Past month e-cigarette use across Census Divisions

Figure 1 depicts the geographical distribution of ENDS consumption across CDs, shedding light on sub-provincial differences in ENDS use rates. The figure shows that even within provinces exhibiting modest ENDS usage rates, the prevalence of vaping varies considerably across CDs. For instance, although Ontario has low documented ENDS use rates, it is home to several CDs where estimated vaping frequencies eclipse the provincial average, like Sudbury District located northwest of Toronto, where the estimated prevalence of past-month vaping exceeds 10% of the population. Similarly, southeastern Ontario’s divisions (e.g., Lennox-Addington and Leeds-Grenville) demonstrate ENDS use rates exceeding the provincial average.

Likewise, select CDs in Quebec bear estimates of past-month ENDS use that surpass the provincial average. The divisions of Acton, Coaticook, Les Sources, and Sherbrooke, positioned to the east of the Montreal metroplex, exhibit higher use rates than other communities in Quebec. In general, though, the estimated frequency of ENDS consumption in most of Quebec’s CDs generally aligns with provincial patterns.

Although Quebec’s overall ENDS consumption is low by national standards, adjacent provinces of New Brunswick, Nova Scotia, and Prince Edward Island show higher use rates—and specific divisions within these provinces appear to drive this trend. While New Brunswick boasts high rates of ENDS consumption, this is mainly attributed to vaping habits in two divisions: Northumberland and Saint John. Similarly, Nova Scotia’s elevated usage is influenced by divisions like Guysborough and Cape Breton. For Prince Edward Island, high vaping rates are observed in Prince County, driving the province’s higher overall ENDS consumption.

Across the nation, distinct patterns emerge with elevated ENDS consumption primarily in suburban, exurban, and rural CDs. For instance, within the Greater Vancouver CD, the estimated past-month rate of ENDS use is under 4%. However, in the adjacent Sunshine Coast district to the north of Vancouver’s urban core, this estimate surpasses 6%. In the central Prairie areas, encompassing Alberta, Saskatchewan, and Manitoba, elevated e-cigarette usage is concentrated in selected divisions, mainly exurban and rural, bordering the USA. Notably, this includes Manitoba’s Division 8—home to the Sandy Bay and Long Plain First Nations.

**Fig. 2:**
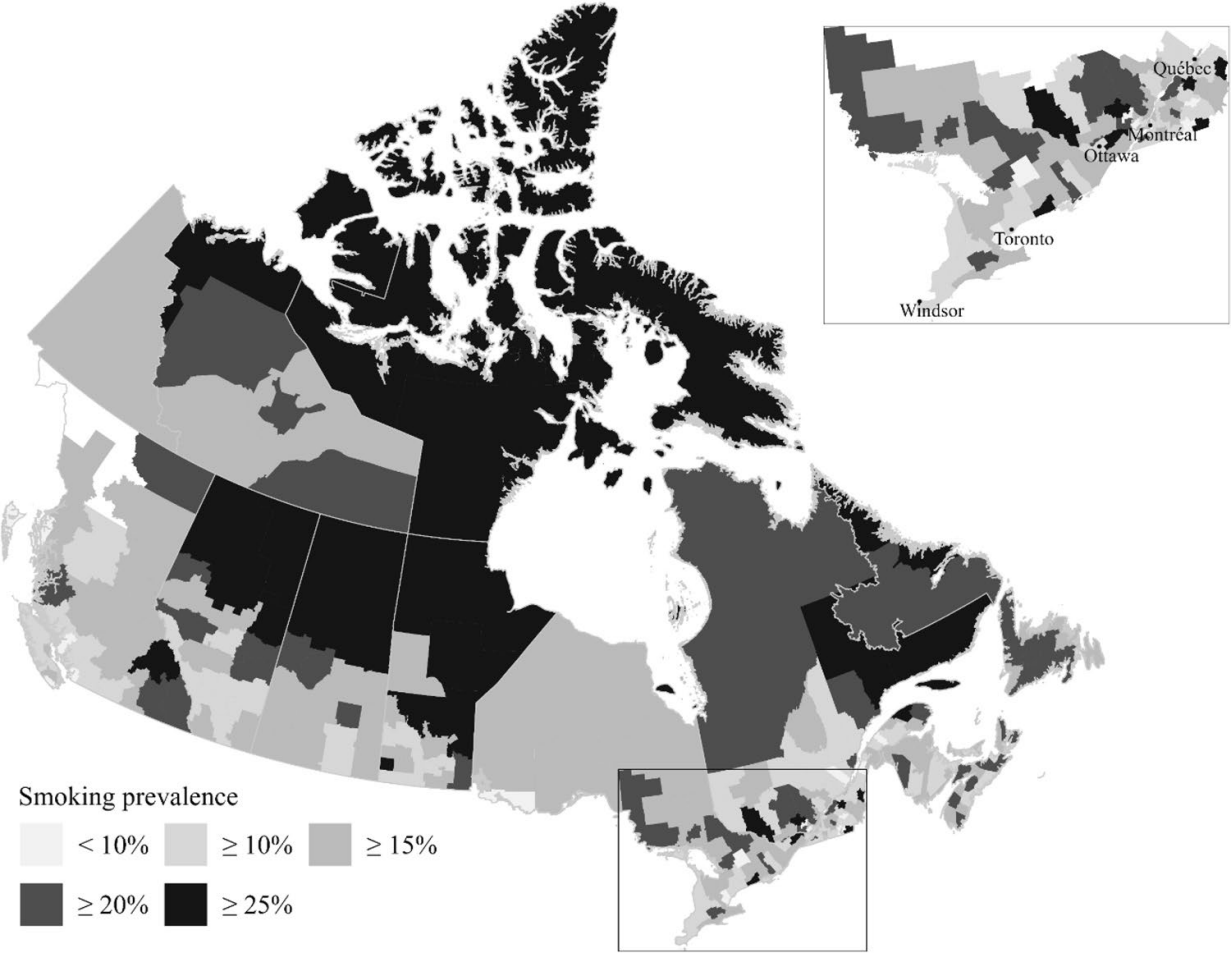
Cigarette smoking prevalence across Census Divisions

At the CD level, there is an inconsistent pattern linking traditional cigarette use and electronic cigarette usage. Figure 2 shows that within the densely populated corridors and main cities of Ontario and Quebec, several divisions with high rates of traditional smoking exhibit low estimated vaping frequencies (e.g., Les Etchemins, Northumberland). Conversely, high estimated rates of *both* smoking and vaping are observed in other divisions (e.g., Coaticook, Lennox, and Addington). The inconsistent association between estimated smoking and ENDS use rates evidenced by the maps warrants further empirical investigation.

**Fig. 3:**
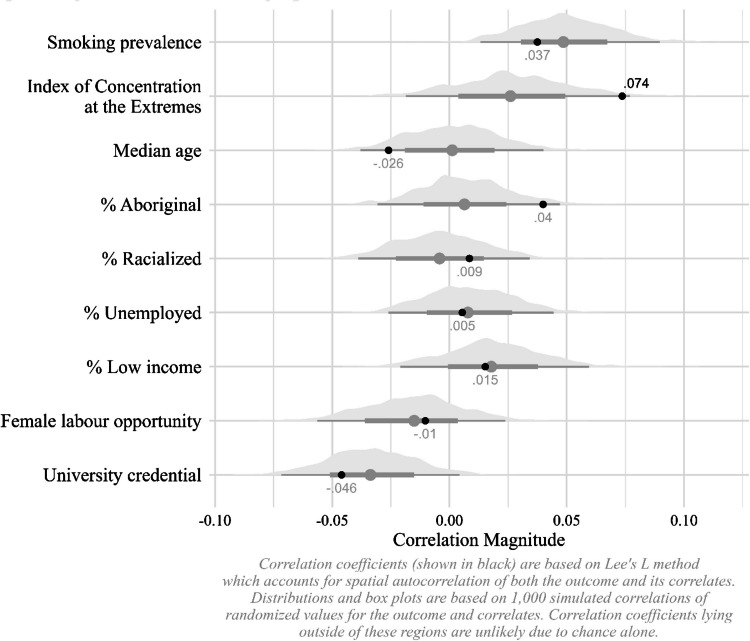
Spatial correlations of vaping with area-level traits

Figure 3 presents spatially explicit correlation coefficients between past month vaping prevalence and community correlates, including estimated smoking rates. The first correlation coefficient shown describes the association between the prevalence of smoking and vaping, producing a Lee’s *L* value of 0.037 (*p* > 0.05)—suggesting that on average the association between estimated vaping and smoking rates is non significant across the 293 CDs under study. Regarding the remaining correlation coefficients, when accounting for the spatial autocorrelation of both vaping and its potential covariates, associations were non significant with one exception. In CDs with higher ICE values—an indicator of concentrated wealth or disadvantage—e-cigarette usage rates were also slightly higher, as evidenced by a Lee’s *L* value of 0.074 (*p* < 0.05). This indicates that vaping may be higher in communities with greater concentrated wealth.

## Discussion

Since electronic nicotine delivery systems (ENDS) arrived in North America, there has been a marked increase in their popularity and accessibility. Implications for youth nicotine uptake are particularly concerning and empirically warranted (Statistics Canada, 2022a). Despite the importance of the broader community context to population health, there are no extant datasets providing ecological measures of smoking and ENDS use at the sub-provincial level. Thus, our study was designed to deepen current understandings of nicotine use across Canadian communities by utilizing precision-weighted estimation (PWE) from multilevel models combining survey data from multiple large-scale studies. Spatial analysis of these empirically estimated data for all 293 Canadian Census Divisions and measures drawn from the Canadian Census reveals two important findings that could usefully inform public policy to reduce nicotine use—especially among Canadian youth.

First, our results show marked geographical variation in ENDS use across Canada. Notably, while provinces like Ontario showed modest overall ENDS use rates, some of its CDs had frequencies exceeding 20%. This contrasts with provincial-level data from the 2017 CTADS, which suggested that Ontario had the lowest ENDS use rates among youth and young adults, at 3.6%. While this encouraging statistic may be trustworthy, it conceals intra-provincial variation in the outcome and obscures geographic variability in need of targeted interventions.

Second, our findings highlight inconsistent associations between traditional and electronic cigarette usage at the CD level. This finding was somewhat surprising, given the strong association between vaping and future smoking identified in person-level longitudinal studies (Yoong et al., 2021). At an ecological level, this association was not observed except for select Census Divisions. This pattern may indicate that while some communities are successfully reducing traditional cigarette use, they face challenges with rising ENDS consumption—consistent with evidence showing that youth and young adults are increasingly initiating nicotine use through vaping.

Beyond the null association between estimated smoking and vaping rates, the spatially explicit correlation coefficients presented earlier reveal few other associations of interest. Notably, only the association between CDs with higher ICE values (an indicator of concentrated wealth) and e-cigarette usage rates was significant. We interpret this finding through the theoretical lens of the diffusion of innovations theory (Rogers, 2003), which holds that the permeation of novel behaviours and products throughout society unfolds sequentially across groups differentiated by the capacity to adopt innovations. Members of privileged communities are better positioned for early adoption of novel behaviours and technologies due to superior access to discretionary capital and digital connectivity—a driver of youth-directed e-cigarette advertising (Gentzke et al., 2022)—as well as permissive social norms that may undermine institutional sanctions on unwanted youth behaviours like nicotine use (Owens, 2022). Our findings align with this premise, though recent data from the USA signals that the diffusion of vaping may have reached “laggards”—groups that presented low propensities for vaping earlier in the diffusion cycle (Jamal et al., 2024). Future research will be needed to track subgroup trends as the capacity to adopt novel forms of nicotine use expands.

In light of the study’s aims, the assembled data resource and analysis provide insights for policymakers, public health officials, and researchers. The observed sub-provincial variation in vaping behaviours can guide targeted prevention and harm reduction efforts in communities with higher rates of use. Additionally, our findings shed light on the implications and efficacy of varying policies and regulations across Canadian provinces, emphasizing the need for a coordinated and informed approach to nicotine regulation. The static nature of the investigation is a weakness, particularly in light of the rapidly evolving landscape of nicotine products. To assess the contemporary relevance of our findings, data from additional rounds of CCHS (2021–2023) were added to our empirical models. Predicted CD-level rates of e-cigarette use with and without this addition were strongly correlated (*r* = 0.94 to 0.99). Nevertheless, future efforts should be made to refresh the data presented here as the balance of nicotine use risks and restrictions evolve.

We considered several additional limitations. First, while PWE and multilevel regression models enhanced the precision of our estimates, potential sources of bias include the reliance on survey data, which may be prone to inaccuracies. Additionally, while our study sheds light on spatial patterns of ENDS use and its correlates, causative relationships must be determined. Further, using Census Divisions as approximations of “communities” may be imprecise. Future research should consider multiple spatial scales. Importantly, the analyses described here are cross-sectional and do not establish causal processes connecting community characteristics to aggregate nicotine use, nor do they distinguish between harm reduction in older smokers and nicotine initiation in youth, masking important age-related differences in e-cigarette use.

While ENDS were initially hailed as a potentially less harmful alternative to traditional tobacco products, their rising popularity, especially among youth, necessitates a comprehensive understanding of their use patterns. By highlighting sub-provincial variations and associated community characteristics, this study provides direction for future research and policy directions aimed at curbing adverse population health implications linked to nicotine use.

## Contributions to knowledge

What does this study add to existing knowledge?

- The study highlights sub-provincial patterns in cigarette smoking and electronic cigarette (“e-cigarette”) usage, demonstrating wide variation in nicotine use between communities within shared regions.

What are the key implications for public health interventions, practice, or policy?

- Continued surveillance of community-level nicotine use is needed to align policies and regulations with the public health goal of nicotine prevention

## Appendix

Table 1

**Table 1:** Data source description

Data source	Target population	Exclusions	Cycles used	Response rate (%)	Link to documentation
Canadian Tobacco Alcohol and Drug Survey (CTADS)	Canadians aged 15 and older in the provinces	Residents of the territories, collective dwellings, reserves, and institutionalized individuals	2013 2015 2017	77.1% 69.8% 70.4%	CTADS documentation
Canadian Tobacco and Nicotine Survey (CTNS)	Canadians aged 15 and older in the provinces	Residents of the territories, collective dwellings, reserves, and institutionalized individuals	2019 2020 2021	44% 41% 42%	CTNS documentation
Canadian Alcohol and Drugs Survey (CADS)	Canadians aged 15 and older in the provinces	Residents of the territories, collective dwellings, reserves, and institutionalized individuals	2019	50.70%	CADS documentation
Canadian Community Health Survey (CCHS)	Canadians aged 12 and older in the provinces	Residents of reserves, Crown Lands, institutions, full-time Armed Forces, isolated areas	2015 2016 2017 2018 2019	59.5% 61.3% 59.5% 61.2% 59.5%	CCHS documentation

## Declarations

Ethics approval was obtained by the University of Ottawa Office of Research Ethics & Integrity.
